# Acylase-Based Coatings on Sandblasted Polydimethylsiloxane-Based Materials for Antimicrobial Applications

**DOI:** 10.3390/polym17020182

**Published:** 2025-01-14

**Authors:** Cláudia A. Silva, Joana Moreira, Marta Fernandes, Andrea Zille, Vanessa F. Cardoso, Md Julker Nine, Filipe S. Silva, Margarida M. Fernandes

**Affiliations:** 1 Center for Micro-Electro Mechanical Systems (CMEMS), Campus Azurém, University of Minho, 4800-058 Guimarães, Portugal; 2 LABBELS-Associate Laboratory in Biotechnology and Bioengineering and Microelectromechanical Systems, University of Minho, 4710-057 Braga, Portugal; 3 Physics Centre of Minho and Porto Universities (CF-UM-UP), Campus Gualtar, University of Minho, 4710-057 Braga, Portugal; 4 LaPMET—Laboratory of Physics for Materials and Emergent Technologies, University of Minho, 4710-057 Braga, Portugal; 5 Centre of Chemistry, Campus Gualtar, University of Minho, 4710-053 Braga, Portugal; 6 2C2T—Centre for Textile Science and Technology, Campus Azurém, University of Minho, 4800-058 Guimarães, Portugal; 7 School of Chemical Engineering, The University of Adelaide, Adelaide, SA 5000, Australia

**Keywords:** quorum sensing, acylase, quorum quenching, PDMS, sandblasting

## Abstract

Indwelling medical devices, such as urinary catheters, often experience bacterial colonization, forming biofilms that resist antibiotics and the host’s immune defenses through quorum sensing (QS), a chemical communication system. This study explores the development of antimicrobial coatings by immobilizing acylase, a quorum-quenching enzyme, on sandblasted polydimethylsiloxane (PDMS) surfaces. PDMS, commonly used in medical devices, was sandblasted to increase its surface roughness, enhancing acylase attachment. FTIR analysis confirmed that acylase retained its three-dimensional structure upon immobilization, preserving its enzymatic activity. The antibacterial efficacy of the coatings was tested against *Pseudomonas aeruginosa* (*P. aeruginosa*) (a common biofilm-forming pathogen), *Staphylococcus aureus* (*S. aureus*), and *Escherichia coli* (*E. coli*). The results showed that sandblasted PDMS surfaces had improved bacterial adhesion due to increased focal adhesion points, but acylase-functionalized surfaces had significantly reduced bacterial attachment and biofilm formation. Notably, the coatings inhibited *P. aeruginosa* growth by 40% under static conditions, demonstrating the potential of acylase-functionalized PDMS for medical applications. This approach offers a promising strategy for creating antimicrobial surfaces that prevent biofilm-related infections in urinary catheters and other medical devices. The findings highlight the dual role of surface roughness in enhancing enzyme attachment while reducing bacterial adhesion through effective QS inhibition.

## 1. Introduction

Biofilms developing on medical devices are a leading cause of nosocomial infections, which significantly contribute to patient mortality and morbidity in healthcare environments. These biofilms are implicated in over 60% of bacterial infections managed in hospitals, with catheter-associated urinary tract infections (CAUTIs) alone accounting for nearly 40% of all hospital-acquired infections. The prevalence of CAUTIs poses a critical health challenge, as they prolong hospital stays, heighten patient trauma, and drive up healthcare expenses.

Understanding the diversity and physiology of the roles that bacteria play is of utmost importance to address these types of challenges, and also antibiotic resistance and infectious diseases in clinical applications. Antibiotic resistance is making the treatment of bacterial infections increasingly challenging, as bacteria are progressively acquiring the ability to withstand the effects of antimicrobial agents [1]. Antibiotic resistance has intensified the need for innovative approaches, such as antibacterial coatings, to prevent bacterial infections and reduce reliance on traditional antimicrobial agents. Thus, antibacterial coatings are designed to kill or inhibit the growth of microorganisms on different types of surfaces [2]. For example, PDMS is commonly used to develop indwelling medical devices such as urinary catheters, which are often prone to microbial contamination and biofilm formation, despite their known intrinsic hydrophobicity and resistance to bacterial adherence. After prolonged usage, these materials still fail to overcome bacterial colonization [3].

**Figure 1:**
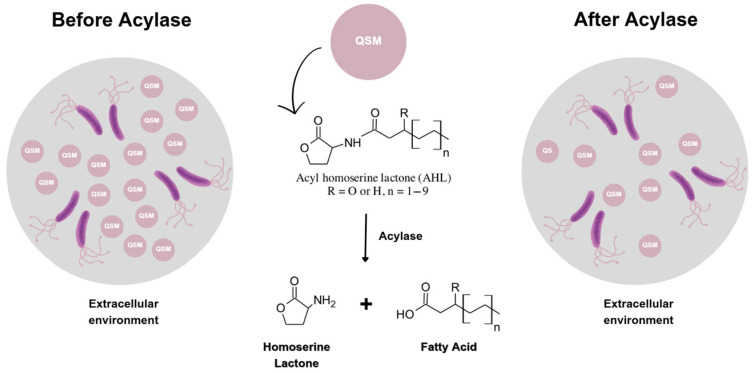
Disruption of quorum sensing in bacteria by acylase. Acylase acts as a QQ agent by cleaving the amide bond in AHLs, the QSM used by Gram-negative bacteria to communicate. By breaking down AHLs in the extracellular environment, acylase prevents these molecules from reaching the threshold concentration needed to activate QS receptors. This inhibition blocks QS-regulated behaviors, such as biofilm formation and virulence factor production, effectively disrupting bacterial communication and reducing infection potential.

One of the mechanisms that regulate biofilm formation is quorum sensing (QS) [4], which allows bacterial cells to communicate and coordinate behavior based on population density [5]. After surface attachment, the bacteria secrete autoinducers (AIs) to the extracellular environment. Once these AIs reach threshold concentrations, they bind to and activate specific receptors, triggering QS-regulated gene expression and initiating biofilm formation. Disrupting this process offers new antibiofilm strategies that may help reduce the risk of resistance development [6]. Whereas conventional antibiotics exert substantial evolutionary stress on bacterial populations by targeting different intracellular components, the QS inhibitors use other bactericidal mechanisms, which induce less selective pressure and therefore decrease the emergence of drug resistance [7]. Inactivation of the AIs in the extracellular environment could provide control of the QS-regulated behaviors and eliminate the need for internalization of the active agents in bacterial cells. Acylase has been reported as a quorum quenching (QQ) enzyme [8] that cleaves the amide bond of acyl-homoserine lactones (AHLs) [9], the QS signals produced, in the extracellular environment, by Gram-negative bacteria (Figure 1). This enzyme was reported to prevent the formation of *P. aeruginosa* biofilm on polystyrene surfaces, interrupting bacterial communication [10].

Antimicrobial coatings comprised of QQ compounds such as acylase could replace the traditional administration of antibiotics in susceptible patients [11]. Techniques such as layer-by-layer [12], sonochemical deposition [13], electrospinning [14], solvent casting [15], and spray coating [16] have been used to develop coatings for increasing the performance of indwelling medical devices. Nevertheless, these techniques use harsh solvents that induce the denaturation of proteins/enzymes or induce a non-homogeneous coating. The challenges in building up QQ-enzyme-based coatings are linked to functional stability (protein conformation) between the coating and target substrate while aiming to reduced bioadhesion between the extracellular secretion and the substrate.

Herein, we propose the application of a simple sandblast technique that uses an abrasive machining [17] on PDMS, to facilitate the creation of micro-roughness with a high surface area, to mechanically lock acylase and obtain a conformationally stable acylase-based antibacterial coating. The functionalized acylase on high-surface-area PDMS is expected to effectively degrade bacterial signaling molecules, thereby inhibiting bacterial colonization and biofilm formation [18].

Unlike traditional methods, acylase has extracellular targets that will not kill bacteria and thus will not contribute to enhancing bacterial resistance [12]. Therefore, it is important to mention that while this work is seeking effective coating build-up on the rough surface, the surface actively reduces bacterial proliferation and bioadhesion. The efficacy of the coatings will be further assessed for their antimicrobial properties, as well as their morphological and physical–chemical characterization.

## 2. Materials and Methods

### 2.1. Enzymes, Material and Bacterium

As a quorum-quenching enzyme, acylase from *Aspergillus melleus* with a protein content of 4.5% (*w*/*w*) and specific activity of 0.05 U·mg^−1^ was acquired from Sigma-Aldrich, Madrid, Spain. Three types of bacteria were studied, two Gram-negative bacteria and one Gram-positive bacteria, respectively. The bacterial strains *P. aeruginosa* CIP 76110, an efficient bacterium in biofilm formation, *E. coli* CIP 110067 and *S. aureus* CIP 7625 were purchased from the Institute Pasteur Collection, Paris, France and used in the bacterial assays. PDMS materials were developed using a two-component kit (a prepolymer and a curing agent; default mixing weight ratio 10:1) of a commercially available silicone elastomer, the SYLGARDTM 184 Silicone Elastomer Kit (Dow, Midland, MI, USA).

### 2.2. Processing of Materials

#### 2.2.1. PDMS Development and Sandblasting Treatment

The PDMS samples were prepared by mixing silicone prepolymer with a curing agent in a weight ratio of 10:1, as recommended by the manufacturer. The mixture was stirred and placed under a vacuum to eliminate the air bubbles present in the mixture. Then, it was carefully cast into a rectangular mold and cured for 2 h at 80 °C. Further sandblasting treatment on PDMS was carried out with a Twin-Pen Sandblaster (Vevor, Lisbon, Portugal). Sandblasting treatment was carried out by industrial sandblasting equipment. The specimens were subjected to blasting by Al_2_O_3_ particles with 53 to 90 µm and 212 to 300 µm diameter at a velocity of 5 m/s and a holding time of 1 min during the sandblasting process. The pressure of the compressed air was about 300 Pa.

#### 2.2.2. PDMS Functionalization

**Figure 2:**
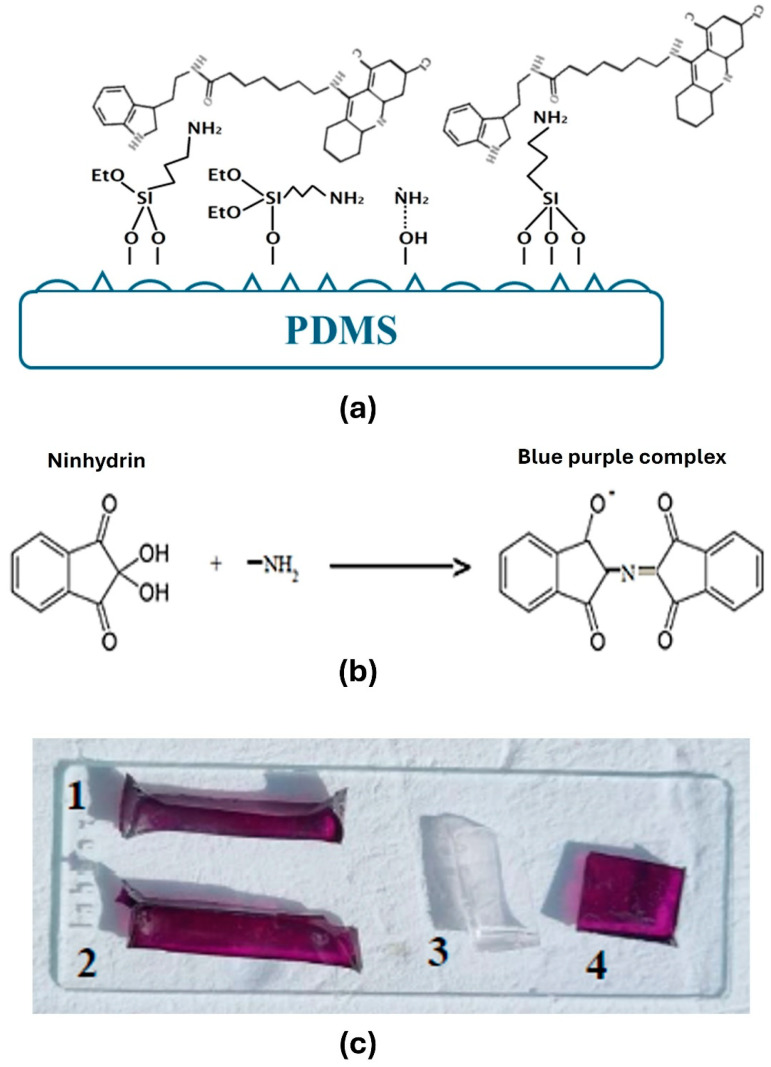
(**a**) Assembly of acylase on the PDMS sandblasted stripes, (**b**) reaction of the ninhydrin test: the amino groups present on the PDMS surface from APTES treatment with ninhydrin will form a blue purple complex, (**c**) ninhydrin test: (1) mesh 180 with APTES; (2) PDMS with APTES; (3) PDMS; (4) PDMS with APTES and acylase.

Prior to coating, the PDMS samples were previously washed with 0.5% (*w*/*v*) sodium dodecyl sulphate (SDS, Sigma-Aldrich) for 30 min, and afterwards, they were rinsed alternately with distilled H_2_O and 96% EtOH and dried with a flow of air. To enhance enzyme attachment, amino groups were introduced onto the surface of the PDMS stripes by pre-treating them with a 5% (*v*/*v*) solution of amino-propyl-tri-ethoxy-silane (APTES, Sigma-Aldrich) (Figure 2a). To confirm the presence of the amino groups onto the surface, the stripes were immersed into a ninhydrin solution (2% (*w*/*v*), Sigma-Aldrich). The APTES-treated PDMS stripes were coated with acylase by dipping them in 1 mg·mL^−1^ acylase solution in tricine buffer pH 8, for 10 min. After acylase deposition, the stripes were subjected to a 10-min rinsing step in 0.15 M sodium chloride (NaCl) solution at pH 8. The samples were dried with a continuous flow of air (flow chamber).

### 2.3. Characterization of the Materials

#### 2.3.1. Attenuated Total Reflection Fourier Transform Infrared Spectroscopy (FTIR-ATR) Analysis

To analyze the deposition of acylase on the PDMS surface, a Nicolet Avatar 360 FT-IR spectrophotometer (Madison, WI, USA) was used to record the spectra. The FT-IR spectra of the two conditions (control and functionalized with acylase) were collected in the attenuated total reflection mode (ATR) at a spectral resolution of 4 cm^−1^, with 45 scans, over the range of 400–4000 cm^−1^ at room temperature. Deconvolution of amide I, II and III band regions was performed using OriginPro 8.5 software. The number of components and their peak position were determined from the second derivative spectrum of the same region. The secondary structure content was calculated from the areas of the assigned peak as a percentage fraction of the total area of the amides I, II and III range. For all data, a linear baseline was fitted, and a smoothing of 15 points with the Savitzky–Golay method was applied.

#### 2.3.2. Water Contact Angle Measurements

The surface wettability of the stripes was assessed by measuring the static contact angle of a 5 µL water droplet using the sessile drop method with a Dataphysics instrument and OCA20 software (version 1.5, Dataphysics, Filderstadt, Germany). Three independent measurements were taken for each sample.

#### 2.3.3. Surface Roughness Measurements

Surface roughness measurements were performed using Mitutoyo 2.0 equipment to evaluate the different sandblasting treatments on the roughness of the PDMS stripes. The average roughness (R_a_) and the difference between the highest peak and lowest valley (R_z_) values were recorded for each sample, providing a quantitative assessment of surface texture. Increased roughness values correspond to a more uneven surface, which can affect properties such as adhesion and wettability. A total of three measurements were performed per sample, and the mean values were reported.

#### 2.3.4. Ninhydrin Test

The presence of amino groups was assessed using the ninhydrin test. Ninhydrin reacts in the presence of amino groups, forming a blue–purple complex, as shown in Figure 2b. The samples were immersed in 2% (*w*/*v*) ninhydrin (Sigma-Aldrich) solution.

### 2.4. Antimicrobial Assays

#### 2.4.1. Initial Bacterial Adhesion

Initial bacterial adhesion on the enzyme-coated and control samples was studied against *P. aeruginosa*, *S. aureus* and *E. coli* strains. The samples were cut into small pieces (0.5 × 0.5 cm) and placed on a 24-well plate. *P. aeruginosa*, *S. aureus* and *E. coli* strains were grown overnight in tryptic soy broth (TSB) at 37 °C and diluted to an optical density OD600 = 0.1. The samples were inoculated with 1 mL of the diluted bacteria and incubated for 2 h at 37 °C to allow bacterial adhesion. After 2 h, the suspension was serial diluted to 10^−7^ and plated on tryptic soy agar (TSA) plates and incubated for 24 h at 37 °C. The colonies were counted and compared against the ones from the control samples.

#### 2.4.2. Bacterial Viability

Two different methods were used to evaluate the antimicrobial properties of the samples after incubation. First, the Live/Dead kit qualitatively assessed the viability of bacteria adhered to the material surface. This was complemented by the OD determination of bacteria in suspension over the material. In parallel, the antibiofilm activity of the enzyme-coated and control samples under static conditions was assessed against *P. aeruginosa* strains using the colony-forming unit (CFU) method. Antibiofilm activity of the enzyme-coating and control samples in static conditions was assessed against *P. aeruginosa* strains using the CFU method. *P. aeruginosa* inoculum was prepared from overnight cultures in LB (Grisp). The samples were cut into small pieces (0.5 × 0.5 cm) and placed on a 24-well plate. Afterwards, 1 mL of the inoculum diluted to an OD600 = 0.1 was added to each well, and the plate was incubated for 24 h at 37 °C. The liquid medium was serial diluted to 10^−7^, plated on LB-agar plates and incubated for 24 h at 37 °C. The corresponding colonies were counted and compared to those on the control sample, where the enzyme was absent. For the biofilm inhibition assays, bacterial cells were aliquoted from a single culture for all replicates of the *Pseudomonas* strain. A LIVE/DEAD BacLight Bacterial Viability Kit for microscopy (Invitrogen, Carlsbad, CA, USA) was used to study the adhesion of bacteria to the surface of the films after incubation without and with magnetic field application. Previously, the samples were washed with phosphate-buffered saline (PBS), followed by staining with a mixture of 1.5 µL green-fluorescent SYTO 9 and 1.5 µL red-fluorescent propidium iodide in PBS for 15 min in the absence of light. Finally, the imaging of the samples was analyzed using a fluorescence microscope (Olympus BX63F2, Olympus, Coimbra, Portugal). The representative fields were captured using a magnification of 100×.

#### 2.4.3. Pyocyanin Quantification

Enzyme-coated and control samples were incubated with an OD600 = 0.1 of *P. aeruginosa* in LB for 24 h at 37 °C. In order to extract the pyocyanin, 0.5 mL chloroform was added to 1 mL cultured supernatant. The samples were then centrifuged for 5 min at 4500 rpm to separate the aqueous phase from the organic phase. To quantify pyocyanin levels, the absorbance of the organic phase was measured at 695 nm (ϵ = 5816 M^−1^ cm^−1^), after centrifugation. For the negative control, *E. coli* was used since the bacterium does not secrete pyocyanin.

#### 2.4.4. Data Analysis

The results are presented as the average of individual measurements with the respective standard deviations, as analyzed by GraphPad Prism Version 9.0.0 for Windows (Graph Pad Software, San Diego, CA, USA). To determine the statistical significance, one-way analysis of variance (ANOVA) was used, followed by the unpaired two-tailed Student’s *t*-test method.

## 3. Results and Discussion

### 3.1. Morphological Characterization

The sandblasting technique was applied on the PDMS surface to increase the superficial area for acylase attachment and to further exert antibacterial and antibiofilm activity. Acylase was immobilized on the PDMS surface in order to obtain a coating with quorum quenching activity. The PDMS surface suffered a pre-amination with APTES, making its amino groups accessible for a better deposition of the acylase layer. The surface of the PDMS contains hydroxyl groups, which, in conjunction with APTES, form an amination reaction [19]. To qualitatively assess the presence of the amino groups on the surface of PDMS, the ninhydrin test was performed. As can be seen from the results (Figure 2c), the samples that underwent APTES treatment showed a characteristic purple color, confirming the presence of NH_2_ groups.

**Figure 3:**
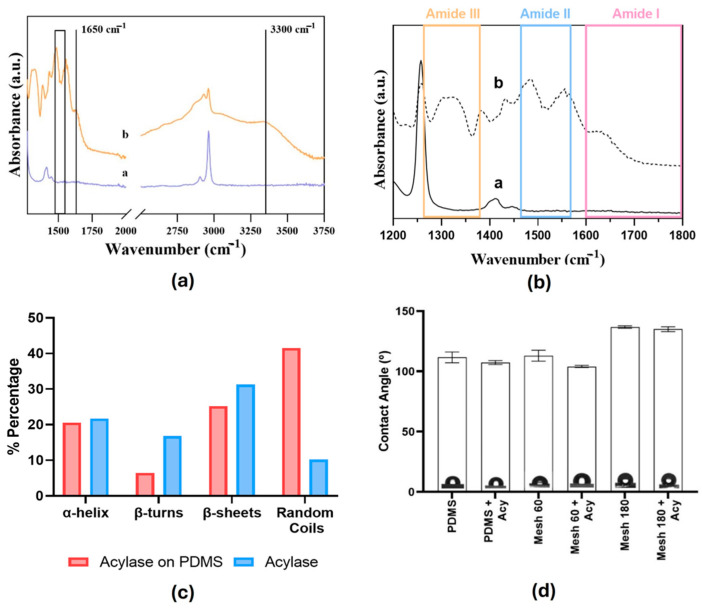
Characterization of the PDMS materials: (**a**) FTIR-ATR spectra of an untreated sample and b acylase-coated sample, (**b**) FTIR spectrum of the materials between 1200 cm⁻^1^ and 1800 cm⁻^1^, highlighting the amide I, II, and III regions. a, untreated sample and b acylase-coated sample, (**c**) quantification of secondary structures in the materials based on FTIR spectral analysis. The graph displays the percentages of α-helix, β-turns, β-sheets and random coils present in both samples; (**d**) surface wettability of PDMS materials before and after treatment (sandblasting and acylase-coating).

After immersion of the PDMS samples in the enzyme solution, typical protein bands were found, which were not observed on the untreated PDMS samples (Figure 3a). The acylase layer was confirmed through FTIR measurements. The acylase-coated samples showed a band around 1650 cm^−1^ and a broad region between 1490 and 1590 cm^−1^, which was assigned to C=O stretching, confirming the presence of amide I in the samples. A band with a maximum of around 3300 cm^−1^ ascribed to N-H stretching (amide A) vibrations was also observed in the spectra. Taking a closer look at the region between 1200 cm^−1^ and 1800 cm^−1^ (Figure 3b), the presence of peaks can be observed at around 1310 cm^−1^, 1530 cm^−1^ and 1650 cm^−1^, which correspond to the presence of amide III, amide II and amide I [20,21], respectively, on our samples. In both samples, it is possible to observe a peak at around 1260 cm^−1^, which corresponds to silicon-methyl stretching [22] (Si-CH_3_). FTIR analysis verified the successful deposition of the acylase layer on the PDMS surface [23] (Figure 3a).

In order to evaluate the 3D protein structure of acylase on the sandblasted PDMS, several methods have been reported for the structural determination of the secondary structure of the protein [24]. One of the methods is FTIR, using deconvolution of amide I, II and III bands. The main groups of the protein’s secondary structure are α-helix, β-sheets, β-turns and random coils (Figure 3c). The choice of the optimal parameters to deconvolute the FTIR spectra is a subjective step in this type of analysis. In our approach, we selected three or more peaks based on the spectra given by the region of amide I, II and III. In amide I, three peaks were selected: 1625 cm^−1^, which corresponds to a β-sheet, 1641 cm^−1^, which corresponds to random coils, and 1681 cm^−1^, which corresponds to β-turns [25,26]. In amide II, five peaks were identified: at 1484 cm^−1^, indicating aggregation or misfolding of proteins, at 1508 cm^−1^ and 1517 cm^−1^, corresponding to extended β-sheet structures, and β-sheet structures, respectively, and at 1553 cm^−1^ and 1558 cm^−1^, corresponding to α-helix and random coils, respectively [25,26,27,28]. In amide III, three peaks were identified, at 1258 cm^−1^, 1297 cm^−1^ and 1329 cm^−1^, which correspond to random coils, β-turns and α-helix, respectively [28,29]. Acylase FTIR spectra were also deconvoluted according to the same parameters as mentioned before in order to assess the potential changes in acylase conformation after immobilization. The results show similar peaks between the samples coated with acylase and the native acylase, as shown in Table 1 and Figure 3c. The percentages of α-helix and β-sheet structures in coated acylase and acylase are very similar in both samples, and it can be concluded that the procedure used to develop the materials does not affect the conformation of the protein.

**Figure 4:**
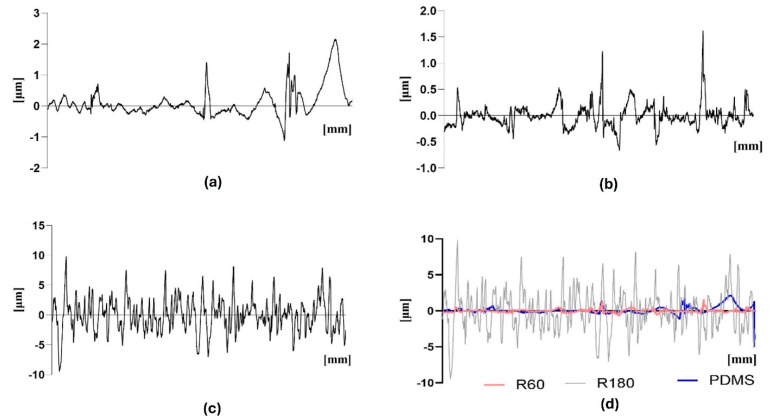
Surface roughness measurements of the materials: (**a**) PDMS, (**b**) mesh 60, (**c**) mesh 180, (**d**) combined.

Differences on the surfaces of the samples were observed in terms of wettability (Figure 3d) and roughness (Figure 4, Table 2). Since two surface modifications, sandblasting and acylase coating were performed, all samples were tested in terms of contact angle measurements. A hydrophilic surface has a contact angle of less than 90°, while a contact angle greater than 90° indicates a hydrophobic surface [30]. PDMS exhibits a hydrophobic surface [31] due to the lack of functional groups capable of attracting water molecules. Considering the results shown in Figure 3d, the presence of acylase does not significantly affect the hydrophobicity of PDMS. However, the sandblasting treatment imparts hydrophobic properties to the samples. These results are consistent with the effect of sandblasting treatment, which increases the surface area of the samples. Given that PDMS is hydrophobic, the increase in surface area due to sandblasting results in an enhancement of the material’s hydrophobic properties.

As sandblasting primarily impacts the roughness of the material, only samples without acylase coatings were tested. The results indicated that the sandblasting treatment increased the surface roughness compared to the untreated samples, which could enhance the adhesion properties of the coating [32]. From the results shown in Table 1 and in Figure 4, it can be concluded that the sandblasting treatment with mesh 60 was not as effective as mesh 180. The sandblasting treatment with a mesh of 180 caused more roughness of the materials than the 60 mesh. When combining the three graphs, it is possible to note the differences between the samples (Figure 4d).

Surface roughness generally strengthens protein attachment, which is often undesirable as it promotes fouling and facilitates bacterial adhesion to materials. According to Georgakopoulos-Soares et al., smoother surfaces are typically favored when designing antimicrobial materials due to their reduced propensity for microbial colonization [33]. However, such surfaces are less reactive to the attachment of bioactive agents. In contrast, rough surfaces improve interactions, enabling more effective binding of antimicrobial agents [34].

### 3.2. Functionality of the Acylase-Coated Materials

After proving the functionalization of the PDMS stripes with acylase, their functionality in terms of antibiofilm activity was also determined. Since initial bacterial adhesion is a crucial step in the biofilm formation process on surfaces, we examined whether the application of enzyme coating could delay cell adherence.

**Figure 5:**
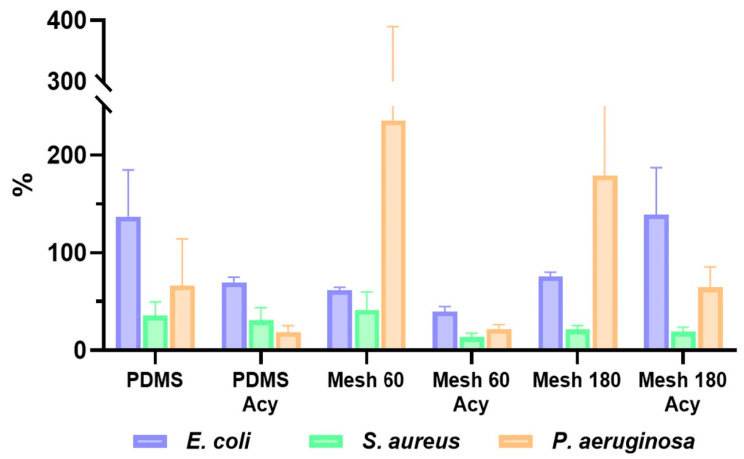
Antimicrobial activity of acylase-coated and untreated samples against *P. aeruginosa*, *S. aureus* and *E. coli* strains in solution after 2 h in contact with material.

As can be seen in Figure 5, the presence of acylase diminishes the bacterial viability. The acylase coating significantly inhibited the initial attachment of *P. aeruginosa* CIP after 2 h of contact, as compared to the untreated silicone material. However, acylase does not significantly affect *S. aureus*, which is in accordance with the literature since acylase is an enzyme that affects the signaling molecules of Gram-negative bacteria. The most significant drop was observed in the mesh 60 sample with *P. aeruginosa*. The results are in accordance with what is found in the literature, since acylase is a quorum-quenching enzyme responsible for degrading AHLs secreted by Gram-negative bacteria, such as *P. aeruginosa* [8].

The rapid increase in cell viability on the sandblasted materials is due to the augmentation of focal hydrophobic adhesion points. As stated in the literature [35,36], hydrophobic interactions are one of the important factors that help promote bacterial cell adherence on surfaces. The primary adhesion of the *Pseudomonas* strain is believed to be governed by the hydrophobic nature of PDMS [12].

These qualitative observations suggest that the development of mature biofilms may be significantly delayed when PDMS surfaces are coated with quorum-quenching enzymes, such as acylase. These findings corroborate the results indicating that acylase coatings effectively disrupt the QS process.

**Figure 6:**
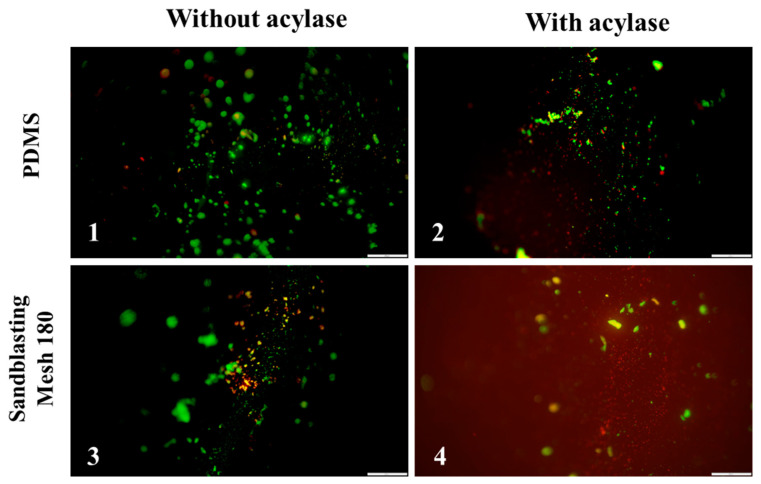
Fluorescence microscopy Live/Dead images for (**1**) PDMS; (**2**) PDMS + acylase; (**3**) mesh 180; (**4**) mesh 180 + acylase; and *E. coli* on different material surfaces after 2 h of incubation. Scale bar represents 20 µm for all images.

Since the roughness of the materials made with sandblasting treatment mesh 60 was not very efficacious, only the mesh 180 and PDMS stripes were used to determine the viability of the bacteria adhering to the surface of these materials. The live/dead assay was employed to evaluate the viability of bacteria following sandblasting treatment and acylase coating treatment. In Figure 6, Live/Dead fluorescent images of *E. coli* are shown, where green fluorescence represents viable cells, whereas red fluorescent cells have compromised cell membranes. The results revealed a decrease in cell viability in the acylase coating group compared to controls, with a higher percentage of red fluorescence indicating increased cell death. These findings prove that the acylase treatment has a cytotoxic effect on bacteria, which is consistent with the literature [9,37,38,39].

**Figure 7:**
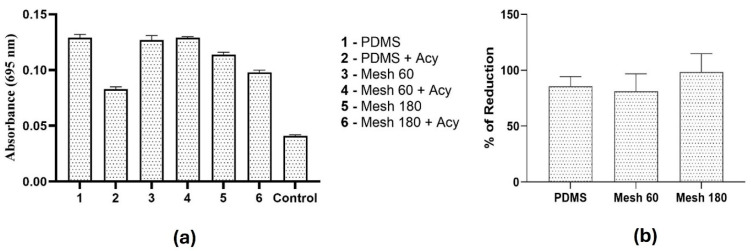
(**a**) Quantification of pyocyanin production by *P. aeruginosa* using UV-Vis spectroscopy at 695 nm. The absorbance values at 695 nm correspond to the concentration of pyocyanin in the bacterial culture supernatant. Samples were measured in triplicate, and error bars represent standard deviations. (**b**) Biofilm production expressed as a percentage relative to the untreated control. Error bars represent standard deviations from triplicate experiments.

The production of pyocyanin was quantified using UV/Vis spectroscopy to verify that the antibiofilm properties of acylase-containing coatings are consistent with a reduction in quorum-sensing activity. *P. aeruginosa* secretes a blue pigment, called pyocyanin, upon induction of quorum sensing [30]. Higher absorbance values indicate increased pyocyanin production, which correlates with enhanced quorum-sensing activity. There is a decrease in pyocyanin secretion by *P. aeruginosa* upon incubation with acylase-containing coatings, as shown in Figure 7a. However, acylase-containing coatings on sandblasted materials do not show a significant decrease in pyocyanin production. Our results corroborate the efficacy of the acylase coatings, offering significant insights into this approach for biofilm inhibition.

*P. aeruginosa* was used to determine the efficiency of the acylase coating materials under static conditions. To determine biofilm inhibition, the area of bacteria was measured and compared to that of the same non-coating acylase material. As demonstrated in Figure 7b, a significant reduction in bacterial growth (80%) was observed across all materials, suggesting that the acylase coatings effectively inhibit biofilm formation by interfering with bacterial communication pathways. Ivanova et al. reported an approximately 80% reduction in *P. aeruginosa* biofilm formation when using acylase layer-by-layer (LbL) coatings on smooth PDMS surfaces [31].

The effectiveness of acylase in inhibiting quorum sensing (QS)-regulated biofilm formation through the in vitro degradation of various model AHL signals has been previously demonstrated [12,40,41]. The enzymatic breakdown of AHLs disrupts bacterial communication, thereby inhibiting bacterial proliferation. The main challenge in this study was ensuring that the protein maintained its active conformation after deposition on a roughened, sandblasted surface.

## 4. Conclusions

The sandblasted PDMS materials developed non-leaching and durable coatings when functionalized with acylase to diminish the viability of bacteria. The analysis confirmed the successful acylase functionalization on the surface modified by sandblasting treatment. An analysis of the antimicrobial tests performed on the stable acylase coatings developed on sandblasted PSMS displayed a significant decrease in bacterial viability, particularly for *P. aeruginosa*, a Gram-negative bacteria present in different environments. Importantly, we proved that materials coated with acylase, even rough materials, offer significant promise for reducing bacterial adhesion. The implications of this work underline the interest in developing antimicrobial surfaces to fight against infections, which continue to present a global health challenge. Future research should focus on more effective and versatile coatings in order to address other surfaces frequently used in the biomedical industry.

## Figures and Tables

**Table 1:** Detailed information on the peak area (%) obtained from the deconvolution of acylase and PDMS coated with acylase.

	Acylase Coating	Peak Area (%)	Molecular Conformation	Acylase	Peak Area (%)	Molecular Conformation
**Amide III**	1258 cm^−1^	24.23%	Random Coils	1252 cm^−1^	5.83%	β—turns
	1297 cm^−1^	18.03%	β—turns	1258 cm^−1^	30.62%	Random Coils
	1329 cm^−1^	57.74%	α—helix	1327 cm^−1^	23.49%	α—helix
				1339 cm^−1^	2.64%	α—helix
**Amide II**	1508 cm^−1^	2.07%		1506 cm^−1^	2.10%	
	1517 cm^−1^	60.24%	β—sheet	1520 cm^−1^	51.73%	β—sheet
	1553 cm^−1^	4%	α—helix	1539 cm^−1^	6.91%	
	1558 cm^−1^	16.85%	Random Coils	1551 cm^−1^	39.25%	α—helix
**Amide I**	1625 cm^−1^	15.26%	β—sheet	1623 cm^−1^	42.03%	β—sheet
	1641 cm^−1^	83.52%	Random Coils	1648 cm^−1^	44.66%	β—turns
	1681 cm^−1^	1.22%	β—turns	1673 cm^−1^	13.31%	

**Table 2:** Surface roughness measurements of the materials. The table shows the difference between the highest peak and lowest valley within the samples (R_z_) and the average roughness (R_a_) values in micrometers (µm).

	PDMS	Mesh 60	Mesh 180
R_z_	1.54 ± 0.19 µm	1.69 ± 0.17 µm	14.71 ± 0.59 µm
R_a_	0.18 ± 0.05 µm	0.21 ± 0.07 µm	2.29 ± 0.22 µm

## Data Availability

Data are contained within the article.
